# Disease of the Respiratory System

**Published:** 1895-08-03

**Authors:** 


### PROGRESS IN MEDICINE.

### DISEASE OF THE RESPIRATORY SYSTEM.

(*Continued from page* 289.)

**The Treatment of Phthisis.**—Fernet has tried direct injection into the lung tissue of solutions of naphthol and of creosote in two hundred cases, choosing especially patients where the disease was localised in an early stage. He claims that it is a rational and safe method of treatment for such patients, though apt to produce temporary cough and pain.[28] F. C. Coley notices the relief from dyspnœa and cough, which soon follows its use. It is an important thing to decide who are really fit patients to send to high altitudes, such as Davos or Colorado. Waxham, after long experience at the latter place, holds it unsuitable for those in whom a large area of lung is involved, or when pyrexia, sweats, and great loss of flesh exist. Nervous cases and those with cardiac complications should avoid the higher altitudes.[29] Solby mentions renal complications as a counter indication, while cases with an hereditary or hæmorrhagic history do well. A third writer, Munn, agrees with Waxham that permanent results can only be looked for if residence there is prolonged for some years, but thinks that cardiac cases do well unless the complication is one directly caused by the lung disease.[30] The importance of diet has been discussed by Soltau, Fenwick, V. D. Harris, and Loomis. The latter advocates in the early stage forced feeding with meat, milk, eggs, and fat six times a day.[31] Harris considers that the dyspepsia is often due to the wasting of the digestive glands as a part of the general marasmus, since the ferments of the pancreas have been found deficient post-mortem in a large proportion of cases, the salivary glands fibrotic, and the saliva in some patients is inactive. Fenwick's account of the fibrotic changes in the stomach glands leads to similar conclusions. Hence much of the dyspepsia is due to the absence of ferments, and must be treated by artificial ones.[31] We may notice here the great value of salol in stopping the diarrhœa of phthisis. F. C. Coley found it most efficacious even in small doses of 5 to 20 grains daily.[32] C. H. Williams recommends large injections of linseed tea as succeeding in some most obstinate cases. He points out, too, that feeding is even less important for the treatment of phthisis than a constant supply of fresh air, for certain patients can easily be made to gain weight while the disease is progressing. He strongly advocates some modification of the open air treatment, as seen at Davos, in our south-coast sanitoria, together with exercise in early cases, in which most foreign treatment is deficient.[33] Potain has confirmed the old view of the great value of tannin in up degeneration of the blood.[36] When oxygen is plentiful, filamentous and branched forms of the bacillus have been found, and it is not impossible that the organism as known to us may turn out to be merely a stage in the life-history of some higher fungus.[37] The presence of cellulose in tubercular organs has been confirmed by Nishimura. It appears to be a product of the bacilli, but nothing is known of its pathology.[38] For the detection of bacilli in the sputum or of urinary casts, Metzger's centrifuge machine is invaluable. In two minutes all the cells, bacilli or casts, are separated from the fluid of the sample, and packed together at one end.[39] Spengler has investigated the bacteria of "mixed infection." In 80 per cent. of the cases streptococcus pyogeneus was present, sometimes in a passive state, and more often taking an active share in the disease, as shown by fever of a peculiar type. The diplococcus, the influenza bacillus, and the M. tetragenus were only found in single instances.[40] Elsner describes the following clinical types of it: (1) Lobar pneumonia attacking a latent tubercular area may show no bacilli until the breaking down of tissue commences; (2) pneumonia arising during ordinary phthisis; (3) streptococcus pneumonia with early hæmoptysis; (4) acute bronchopneumonia due to depressing agents during phthisis. However, the bacillus alone may at times produce a pneumonia. Tuberculosis of the bile ducts has been examined by Sergent, who finds Sabourin's view correct that it generally proceeds from without inwards, though the bacillus is unaffected by a sojourn in bile.[42] As perhaps the earliest symptom of phthisis, C. W. Ingraham has directed attention to a rise of a half or one degree of temperature daily before any physical signs have appeared. By six observations a day for a fortnight a positive diagnosis may often be made in the absence of other symptoms.[43]

**Fibroid Diseases of the Lung.**—This condition of the lung, which Sir Andrew Clark brought so prominently before our notice, was recently discussed by Dr. Percy Kidd,[44] in his lectures at the London Hospital. From an ætiological standpoint fibroid diseases of the lungs fall into two main groups—(*a*) Tuberculous cirrhosis, and (*b*) non-tuberculous cirrhosis. Of these groups the first is by far the larger; tubercle, in other words, is infinitely the most common cause of induration of the lung. Dr. Kidd points out that tuberculous cirrhosis is a better term than fibroid phthisis, that introduced by Sir Andrew Clark, as the latter has been made to include non-tubercular cases. Now in every case of chronic induration of the lung there is always a certain amount of fibrosis. Yet for clinical purposes we want to distinguish between ordinary cases of chronic tuberculosis with limited induration, and those in which the fibroid element predominates.

The indurative process in tuberculous cirrhosis, as in other forms of pulmonary fibrosis, travels along preformed lines; there is thickening around the bronchial tubes, between the lobes, between the lobules, and in the alveolar walls, extending sometimes up to the pleura. Thickening and adhesion of the pleura are always present. The tubercular differs from the non-tubercular form in one or two points—viz., in locality, the apex of the lobe being generally the part first affected, in the presence of caseous nodules, and in a tendency to the formation of vomicæ, although the latter is not absolutely characteristic of the tubercular form.

The tubercular process extends in the lungs in the following ways: Firstly, by direct continuity, preferably along lymph channels; secondly, by the formation of fresh foci by tubercle bacilli, which are carried in the lymphatic circulation for some little distance in the lung; thirdly, and most important of all, a cavity having formed in the lung, its secretions escape into the corresponding bronchus, and are expelled by cough. But the discharge of sputum is apt to be incomplete, and their infective material is liable to be sucked back into other bronchi, and into healthy lung tissue, and thus arise fresh centres of disease.

Hereditary predisposition is less common in these cases, perhaps, than in the ordinary forms of tuberculosis, and Dr. Kidd attaches little importance to it from a diagnostic point of view, though it is of considerable importance with regard to prognosis. As a rule, tuberculosis manifests a greater chronicity in old patients than in young, but the history of the fibroid tubercular disease is nearly always a very chronic one, resembling that of chronic bronchitis. The patient has had a chronic cough for years, losing it in summer, but manifesting it in an aggravated form with the return of winter, or the cough is paroxysmal, occurring generally in the morning; while in other cases there is hardly any cough at all, although there may be physical signs of marked disease of the lung. In other cases, recurrent hæmoptysis may be the only symptom noticed by the patient. Hæmoptysis, when it occurs, affects the prospects of the patient very seriously.

The non-tuberculous form of cirrhosis may be divided into three classes—(1) the massive form, which is almost synonymous with the lobar variety; (2) the insular, broncho-pneumonic or patchy form; (3) the reticular form.

In the first or massive form, which involves the whole or part of a lobe, converting it into a solid tissue of varying degree of firmness, with some amount of general contraction, bronchiectasis is not so common as in the next form, but a certain amount of destructive excavation is very frequent. Pulmonary aneurisms are not common. Emphysema is usually present in the other parts of the same lung, and on the opposite side, but fibrous adhesions of the pleura are almost invariably found. The localisation is important from a diagnostic point of view, for whereas in tubercular cirrhosis the process is most marked at the apices, the lower lobes are most affected in the non-tubercular form, which is nearly always confined to one lung.

In the insular form patches of indurated tissue are found irregularly scattered in the lungs, especially towards the bases, and bronchiectasis is very common indeed, and is apt to be sacculated. The reticular form is characterised by a number of intersecting fibrous bands in the lung tissue, giving the appearance of a sort of trellis work. Pigmentation is present, giving the slatey, patchy appearance to the lung. It is casually associated with emphysema and acute phthisis, and in the early stages of chronic. Rabbits, too, were made more resistent to tubercle by its use.[34] Robin has investigated the products of metabolism in phthisis, and finds a great diminution of the urinary solids where the disease is advancing. In the later stages even fever is rarely accompanied by an increase.[35]

**Pathology.**—Angelo Maffucci finds that from the bodies of dead bacilli a toxic product is formed unaffected by light, heat, drying, or time, causing marasmus even when in the most minute doses. It is transmitted to the fœtus from the parent, and sets chronic bronchitis, and during life it is very rarely diagnosed from simple chronic bronchitis.

**Minor Affections.**—Various plans for the treatment of common colds, that reproach of medicine, have been brought forward by different writers. Thus M. Maurel, finding the destructive power of iodoform upon staphylococcus albus, has employed a nasal plug saturated with iodoform during the early stages of a catarrh. He reports rapid cures, and considers that descending bronchitis is prevented.[45] Almost the last work of the late Dujardin-Beaumetz was a paper recommending treatment by a full dose of aconite (repeated if necessary) to cut short a cold[46]; while Marcele Lermoyez recommends fifteen minims each of tincture of aconite and tincture of belladonna in divided doses, or the use of menthol, salol, and cocaine snuff, and carbolic inhalations. The first is the only remedy on which much reliance can be placed as an abortive. To avoid constant recurrence, any adenoids or hypertrophic rhinitis must be treated.[47] The "Colorado Climatologist" advises as an abortive to paint the nostrils, and especially the swollen turbinated bodies, with, first, a 1 per cent. solution of atropine, and then with a 5 per cent. solution of cocaine. The patient can prevent a relapse by the use of a snuff containing cocaine, bismuth and camphor, or morphia used occasionally.[48] For fœtid bronchitis Lancereaux recommends sodium hyposulphite in daily doses not exceeding 60 grains,[49] while Chaplin obtains a temporary cure by causing the patient to breathe an hour daily in the vapour of common tar creosote, which is evolved from a heated dish in a very small room. In some cases a complete purification seems to take place; in others, occasional inhalations are needed to prevent the fœtor returning. The putrefactive bacteria found in the sputa at first were entirely removed by the inhalations.[50] The pathology of a case of gangrene has been studied by Reinbach. An emphysematous state following on pertussis appears to have afforded the conditions needed for the bacilli. These closely resembled b. anthracis, but the exact species was not determined. A number of cavities such as are found in interstitial emphysema were scattered through the upper lobes, and in these cavities, it is thought, the low vitality of the tissues enabled the bacilli to commence growth.[51] Duflocq describes a case of bronchitis with pneumococci in the sputa, and declares that these bronchial cases are always marked by the same insidious onset, grave features, and slow convalescence. In another instance of bronchitis the bacillus coli was present in the sputa after a previous fracture of the humerus. Such cases are of bad prognosis, and their symptoms somewhat resemble those of cholera.[52] There are indeed probably many types of bronchitis of very different degrees of severity which can only be separated by a bacteriological examination. The true explanation of "mountain sickness" has been arrived at from the report of Mr. Conway's explorations in the Himalayas and Professor Roy's papers on them. The symptoms are first felt when a height of 17,000 feet is reached, and consist in dyspnœa fatigue and a feeling of anxiety, with palpitation, &c., passing into paralysis and unconsciousness. The affection is probably of the nature of asphyxia, dependent on the reduced amount of oxygen supplied to the tissues. The pulse tracings do not so far support the view that heart failure is at the bottom of the symptoms.[53]

The important subjects in F. T. Roberts' lectures on combinations of morbid conditions of the chest include among many others a discussion of an affection where the organs show little if any sign of disease, but the thorax is rigid and the muscles are wasted, and which is often labelled simply weak chest. The state is an important one. The patients succumb readily to disease, but live long under favourable conditions. This condition of fixed chest walls and inelastic lungs and feeble heart is well worth noting.[54]

A type of chronic pneumonia, too, is of interest where one lung becomes solid and enlarged, while the opposite lung enlarges also, and is often marked by dry bronchial rales, so that the prominent symptoms are those of emphysema and bronchitis. Laborde's method of rhythmical traction on the tongue as a means of treating asphyxia has been proved valuable in many accidents. The tongue is grasped firmly by a cloth and drawn out sharply eighteen times a minute and as quickly released. The whole body of the tongue must be moved forward. Artificial respiration by pressure on the chest may be carried on by assistants simultaneously. In electrical accidents and injuries to the brain it may be of importance to use both methods of restoring respiration, and to continue them for a long time. The comparative values of this and the older methods have been much discussed.

28 Med. Week, Ap. 5. 29 Ther. Gazette, Ap. 15. 30 Ther. Gazette, Ap. 31 Lanc., Nov. 10. 32 Pract., Oct. 33 Bir. Med. Rev., Dec. 34 Int. Med. Mag., Jan. 35 Med. Week, Ap. 5. 36 B. M. J., Jan. 19. 37 B. M. J., Mar. 9. 38 A. M. S. Bulletin, Feb. 39 Lanc., Dec. 8. 40 Am. J. Med. Sc., Ap. 41 Med. Chur., Mar. 42 Med. Week, Mar. 10 43 Med Rec., May 4. 44 Clin. Jour., Nov. 7. 45 N. Y. Med. Jour., Feb. 9, 1895. 46 Med. Rev., Mar. 23. 47 N. Y. Med. Jour., Feb. 16. 48 A. M. S. Bulletin, Mar. 49 Jour. Des Prac., No. 27, 1894. 50 B. M. J., Nov. 24. 51 Am. J. Med. Sc., Dec. 52 B. M. J., Jan. 19, 1895. 53 B. M. J., Jan. 5. 54 Lanc., Jan. 26. 55 Med. Week, Dec. 7.

